# Investigating the Ca^2+^-dependent and Ca^2+^-independent mechanisms for mammalian cone light adaptation

**DOI:** 10.1038/s41598-018-34073-8

**Published:** 2018-10-26

**Authors:** Frans Vinberg, Vladimir J. Kefalov

**Affiliations:** 1 Ophthalmology and Visual Sciences, Washington University School of Medicine, St. Louis, Missouri USA; 2 Present Address: John A. Moran Eye Center, University of Utah, Salt Lake City, Utah USA

## Abstract

Vision is mediated by two types of photoreceptors: rods, enabling vision in dim light; and cones, which function in bright light. Despite many similarities in the components of their respective phototransduction cascades, rods and cones have distinct sensitivity, response kinetics, and adaptation capacity. Cones are less sensitive and have faster responses than rods. In addition, cones can function over a wide range of light conditions whereas rods saturate in moderately bright light. Calcium plays an important role in regulating phototransduction and light adaptation of rods and cones. Notably, the two dominant Ca^2+^-feedbacks in rods and cones are driven by the identical calcium-binding proteins: guanylyl cyclase activating proteins 1 and 2 (GCAPs), which upregulate the production of cGMP; and recoverin, which regulates the inactivation of visual pigment. Thus, the mechanisms producing the difference in adaptation capacity between rods and cones have remained poorly understood. Using GCAPs/recoverin-deficient mice, we show that mammalian cones possess another Ca^2+^-dependent mechanism promoting light adaptation. Surprisingly, we also find that, unlike in mouse rods, a unique Ca^2+^-independent mechanism contributes to cone light adaptation. Our findings point to two novel adaptation mechanisms in mouse cones that likely contribute to the great adaptation capacity of cones over rods.

## Introduction

Our daytime vision is mediated by cone photoreceptors, which can adapt quickly and over a wide range of ambient light levels. In contrast, rod photoreceptors, which mediate our dim light vision, adapt slower and saturate under bright light. However, the molecular underpinnings explaining the faster and more efficient capability of cones to light adapt, i.e. to regulate their sensitivity in response to increments and decrements of background light, are still poorly understood.

Absorption of a photon by visual pigment molecule triggers a G protein signaling cascade involving identical or homologous rod and cone proteins^1,2^. Briefly, a single photoactivated rod or cone pigment can activate several cell-specific heterotrimeric G proteins, transducins^3–5^. In turn, each of these transducins disinhibits a rod/cone-specific effector enzyme phosphodiesterase, PDE6^6,7^. The resulting upregulated hydrolysis of cGMP by PDE6 leads to a lower cGMP concentration in the outer segments of photoreceptors, followed by closure of cGMP-gated (CNG) channels in their plasma membrane^8^, reduced influx of Na^+^ and Ca^2+^, and ultimately cell hyperpolarization^9,10^. Continuous photoreceptor function requires the inactivation of the visual pigments, transducins and PDE6, and the upregulation of cGMP synthesis by guanylate cyclases (GC) to restore and maintain cGMP concentration^11,12^. The concerted activation and inactivation of these transduction components upon light stimulation is modulated by their properties and expression levels, and produces a light response with distinct rod/cone-specific amplitude and kinetics^13–22^.

When background light levels change, photoreceptors adjust their sensitivity allowing vision even under rapidly changing ambient illumination. Part of this change in sensitivity is driven by the modulation of cGMP turnover^23^. However, the dominant mechanism for modulating photoreceptor sensitivity in background light (referred to below as light adaptation) is thought to be driven by Ca^2+^-dependent feedback on the phototransduction cascade triggered by the decrease in outer segment Ca^2+^ concentration upon light stimulation^24–27^. This feedback is mediated by several Ca^2+^-binding proteins, including 1) Guanylate Cyclase Activating proteins (GCAP1 and GCAP2), which activate guanylyl cyclase and thus accelerate cGMP synthesis in low Ca^2+^^28,29^, 2) recoverin (Rv), which dissociates from rhodopsin kinase (GRK1) in low Ca^2+^ allowing GRK1 to accelerate visual pigment inactivation in both rods and cones^30–34^, and, in the case of amphibian photoreceptors. 3) calmodulin and/or CNG modulin, which modulate the gating of CNG channels^35–37^. Notably, rods and cones share the same GCAPs and recoverin isoforms, leaving the question of the mechanisms that produce the difference in their light adaptation still open.

It has been demonstrated that light adaptation in amphibian rods and cones is mediated by Ca^2+^^26,38^. A recent study showed that the same is likely also true for mammalian rods^39^ but whether mammalian cone light adaptation is mediated exclusively by Ca^2+^-dependent mechanisms is not known. Here we combined electrophysiology, pharmacology, and genetic approaches to dissect the contribution of Ca^2+^-dependent and Ca^2+^-independent mechanisms to the light adaptation capacity of mammalian cones in the absence of the known Ca^2+^ feedbacks mediated by GCAPs and recoverin.

## Methods

### Ethical approval

All experimental protocols were in accordance with the Guide for the Care and Use of Laboratory Animals and were approved by the institutional Animal Studies Committee at Washington University.

### Animals

Mice were housed in the University’s animal facilities under 12/12 hour light/dark cycle and had free access to water and regular rodent chow. The *GCAPs*^−/−^^28^ and *Rv*^−/−^^40^ mice were originally obtained from Dr. Jeannie Chen (University of Southern California) but were backcrossed for several generations to the *Gnat1*^−/−^ background^41^ to allow for cone-specific recordings. We crossed these *GCAPs*^−/−^ *Gnat1*^−/−^ and *Rv*^−/−^ *Gnat1*^−/−^ to produce *GCAPs*^−/−^ *Rv*^−/−^ *Gnat1*^−/−^ mice. We then compared the functional properties of G*nat1*^−/−^ control cones and *GCAPs*^−/−^ *Rv*^−/−^ *Gnat1*^−/−^ cones to evaluate the functional contribution of residual Ca^2+^-dependent and any possible Ca^2+^-independent regulation of cone phototransduction. All tested mice were between 2–3 months of age. Mice were genotyped from tail samples by Transnetyx Inc. and were also tested to be free of *Rd8* mutation^42^.

### *Ex vivo* Electroretinogram (ERG) Experiments

Transretinal ERG responses to flashes and steps of cyan LED light (505 nm Luxeon Rebel LED SR-01-E0070) from isolated mouse retinas were recorded as described previously^43^. Briefly, the retinas were mounted on a specimen holder where they were perfused 2 mL/min with Locke’s solution heated to 37 °C. The perfusion solution contained (in mM): NaCl, 112; KCl, 3.6; MgCl_2_, 2.4; CaCl_2_, 1.2; HEPES, 10; NaHCO_3_, 20; Na_2_-succinate, 3; Na-glutamate, 0.5; glucose, 10. The solution was equilibrated with 95%O_2_/5%CO_2_ at 37 °C. In addition, 2 mM L-Aspartate, 40 μM DL-AP4 (Tocris Biosciences) and 100 μM BaCl_2_ were added to the medium to isolate the photoreceptor component of the ERG signal. Low Ca^2+^ solution was prepared by adding 0.4 mM EGTA and substituting the 1.2 mM CaCl_2_ with only 0.1 mM CaCl_2_ added to the medium, estimated to produce ~30 nM free Ca^2+^ concentration^44^.

Signals were amplified initially by a differential amplifier (DP-311, Warner Instruments) and then amplified further and low-pass filtered at 300 Hz (8-pole Bessel, Krohn-Hite Corporation, model 3382) and sampled at 10 kHz with 0.03 μV resolution by a digitizer (1440 A Digidata, Molecular Devices) and pCLAMP 10 software (Molecular Devices). Light stimulation was provided by a custom-build LED system via optical cable (Newport, 77536) and the optics of an inverted microscope that produced homogenous light over the effective measurement area of ∅0.5 mm at the central retina. Light intensity and the length of light flashes (1 ms) and background light steps were controlled by an LED driver (Thorlabs, LDC210C) and neutral density filters. The total light power of the LED stimuli (λ_max_ at 505 nm; Rebel star, SR-01-E0070) was measured by calibrated optometer (UDT Instruments, Model 211) near the plane of the retina. The intensity was then calculated based on the light spot area at the plane of the retina (∅2.35 mm) and converted to a number of 505 nm photons μm^−2^ s^−1^.

### Analysis

Origin 9.0.0 software (64-bit, SR2, OriginLab) was used for data analysis and figure preparation. A Naka-Rushton function was fitted to the response amplitude (*r*) from G*nat1*^−/−^ control and *GCAPs*^−/−^ *Rv*^−/−^ *Gnat1*^−/−^ mouse cones:

1 $$\frac{r}{{r}_{\max }}=\frac{{I}_{F}}{{I}_{1/2}+{I}_{F}},$$

where *r*_*max*_ is the maximal amplitude of a saturated cone response, *I*_*F*_ is the intensity of the light flash (in photons µm^−2^), and *I*_*1*/*2*_ is the light intensity producing a half-maximal photoresponse.

A modified Weber-Fechner function was fitted to the light adaptation data:

2 $$\frac{{S}_{F}}{{S}_{F,D}}=\frac{{I}_{0}^{n}}{{I}_{0}^{n}+{I}^{n}},$$

where *S*_*F*_ is the sensitivity of cones defined as the amplitude of a response to dim flash divided by the flash strength (in 505 nm photons μm^−2^), *S*_*F*,*D*_ is the sensitivity in darkness defined as the amplitude of a response to dim flash divided by the flash strength with no background light present, *n* is a slope factor, *I* is the background light intensity (in 505 photons μm^−2^ s^−1^) and *I*_0_ is the background light intensity at which the sensitivity S_F_ drops to 50% of that in darkness. In all cases, response amplitude was measured at its peak.

The decline in sensitivity as a function background light intensity in the absence of light adaptation mechanisms was calculated by using two functions. Firstly, the exponential saturation function

3 $$\frac{{s}_{F}}{{s}_{F,D}}={e}^{-\frac{{s}_{F,D}{T}_{i}I}{{r}_{\max }}},$$

where T_i_ is the integration time of a dim flash response defined as the area between the trace and time-axis along the baseline of the response divided by the response peak amplitude and r_*max*_ is the maximal response amplitude of a saturated cone response. Alternatively, a function

4 $$\frac{{s}_{F}}{{s}_{F,D}}=\frac{1}{{(1+\frac{{s}_{F,D}{T}_{i}I}{3{r}_{\max }})}^{4}}$$

that has been derived by removing all the feedbacks from a phototransduction model was used^45^.

## Results

### Cones can light adapt in the absence of GCAPs and recoverin

**Figure 1:**
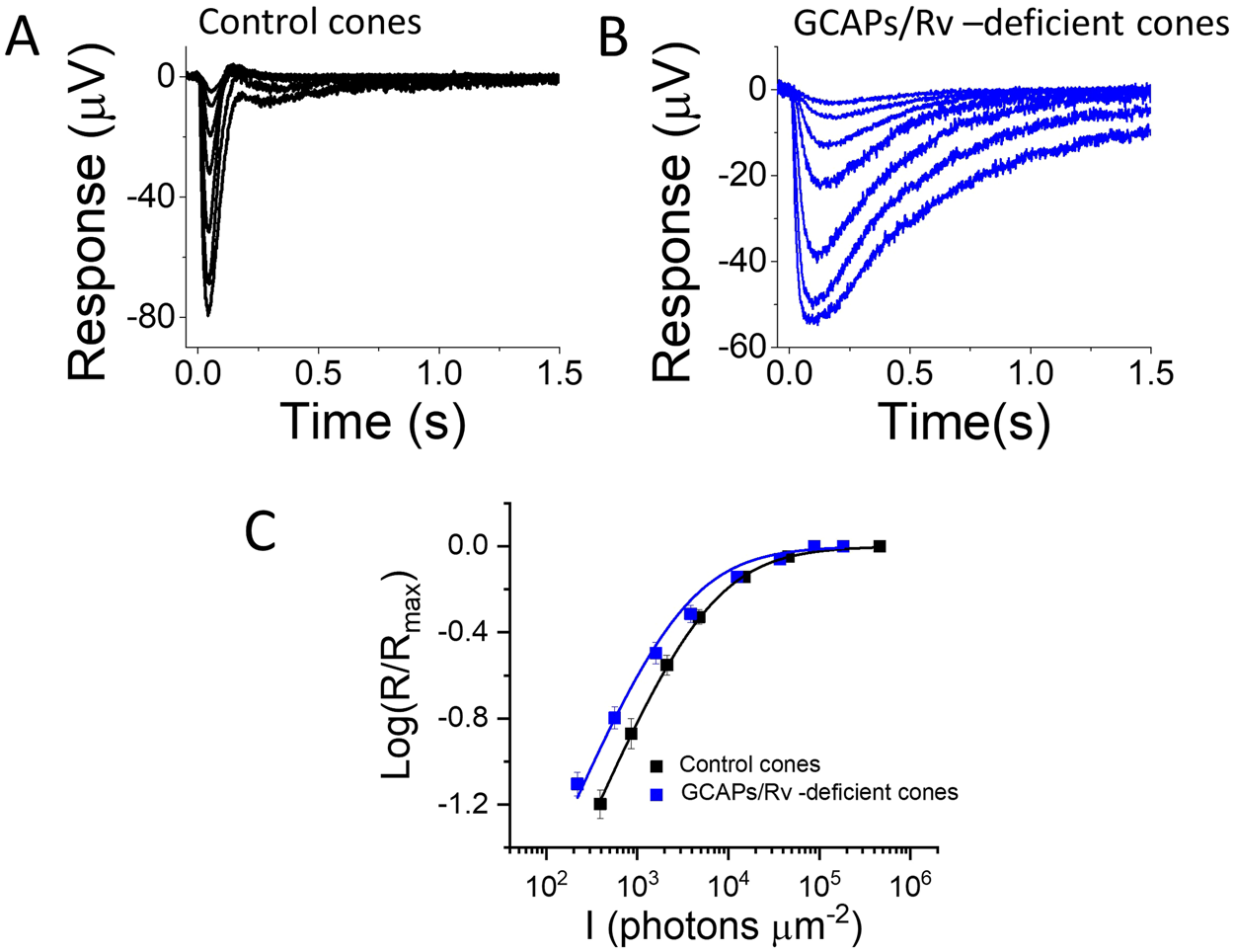
GCAPs/recoverin-deficient cones are more sensitive and have slower photoresponse kinetics. Responses of control (**A**) and GCAPs/recoverin-deficient (**B**) cones to flashes of 505 nm light from 400 to 460,000 and 220 to 180,000 photons μm^−2^ in control *Gnat1*^−/−^ and *GCAPs*^−/−^ *Rv*^−/−^ *Gnat1*^−/−^ mice, respectively. (**C**) Response amplitudes (mean ± SEM) plotted as a function of flash intensity (in photons μm^−2^) for control *Gnat1*^−/−^ (black squares, n = 4 mice) and *GCAPs*^−/−^ *Rv*^−/−^ *Gnat1*^−/−^ (blue squares, n = 4 mice) mice. The smooth lines plot Eq. (1) with *I*_*1*/*2*_ = 5,600 photons μm^−2^ (black) and 3,000 photons μm^−2^ (blue) for control and GCAPs/recoverin-deficient cones, respectively.

The role of GCAP1, GCAP2 and recoverin (Rv) in mouse rod and cone phototransduction and light adaptation has been well characterized^28,31,32,39,46–49^. Here, we bred *GCAPs*^−/−^ (lacking both isoforms 1 and 2) and *Rv*^−/−^ mice to produce *GCAPs*^−/−^ *Rv*^−/−^ double knockout mice. The rods and cones in these mice lack the Ca^2+^ feedbacks to modulate cGMP synthesis (*via* GCAP1/2) and active visual pigment lifetime (*via* recoverin) that are known to contribute to the rod and cone phototransduction termination and light adaptation^28,31,32,46^. To investigate how the simultaneous deletion of GCAPs and recoverin affects the phototransduction in mouse cones, we performed *ex vivo* ERG recordings from dark-adapted isolated retinas (see Methods, Fig. 1). To facilitate assessment of cone physiology, all of the mice were on *Gnat1*^−/−^ background to remove the light responses originating from their rod photoreceptors^41^. We compared the responses from control *Gnat1*^−/−^ cones with the responses from *GCAPs*^−/−^ Rv^−/−^ *Gnat1*^−/−^ cones. The calcium feedback deficiency in *GCAPs*^−/−^ *Rv*^−/−^ *Gnat1*^−/−^ cones resulted in slower shut-off of their photoresponses, leading to larger response integration time (Fig. 1A,B, and Table 1; see also^46^). The larger integration time also appeared to increase the sensitivity of *GCAPs*^−/−^ *Rv*^−/−^ *Gnat1*^−/−^ cones, as demonstrated by an apparent shift of their response amplitude data to dimmer light (Fig. 1C). As a result, the averaged cone light flash intensity required to elicit 50% of the maximal response (*I*_*1*/*2*_) was reduced by the deletion of GCAPs and recoverin (though the difference was not statistically significant, see Table 1). These results are consistent with previous rod studies showing that *GCAPs*^−/−^ *Rv*^−/−^ rods are more sensitive to light and have slower response kinetics as compared to wild type rods^28,40^.

**Table 1:** Parameters of photoresponses from control Gnat1^−/−^ and from GCAPs^−/−^ Rv^−/−^ Gnat1^−/−^ mouse cones in normal and low Ca^2+^.

	*r*_*max*_ (μV)	*I*_*1*/*2*_ (phot. μm^−2^)	*t*_*p*_ (ms)	*T*_*i*_ (ms)	*I*_0_ (phot. μm^−2^ s^−1^)	*n*
Control *Gnat1*^−/−^	56 ± 7	5,500 ± 700	56 ± 2	71 ± 5	48,800 ± 11,000	1.0 ± 0.1
*GCAPs*^−/−^ *Rv*^−/−^ *Gnat1*^−/−^ (normal Ca^2+^)	50 ± 10	3,800 ± 400	142 ± 12*	240 ± 40*	12,100 ± 2,400	1.5 ± 0.1*
*GCAPs*^−/−^ *Rv*^−/^ *Gnat1*^−/*–*^, (low Ca^2+^)	72 ± 9^†^	5,400 ± 1,100^†^	139 ± 10	420 ± 30^†^	42,900 ± 6,400^††^	2.0 ± 0.2^††^

*r*_*max*_, saturated photoresponse amplitude; *I*_*1*/*2*_, light flash intensity producing a half-maximal light response (see Eq. 1); *t*_*p*_, time-to-peak of a dim flash response; *T*_*i*_, integration time of a dim flash response defined as the integrated area between the response and baseline divided by the response amplitude; *I*_0_, background light intensity reducing the sensitivity to 50% of the dark-adapted sensitivity; *n*, slope of the light adaptation curve (see Eq. 2). *p < 0.05 (paired t-test between control Gnat1^−/−^ and GCAPs^−/−^ Rv^−/−^ Gnat1^−/−^ data in normal Ca^2+^); ^†^p < 0.05; ^††^p < 0.01 (paired t-test between normal and low Ca^2+^ data of GCAPs^−/−^ Rv^−/−^ Gnat1^−/−^ mice). Control Gnat1^−/−^ data (mean ± SEM) is from 4 mice, and GCAPs^−/−^ Rv^−/−^ Gnat1^−/−^ data (mean ± SEM) is from 8 mice.

**Figure 2:**
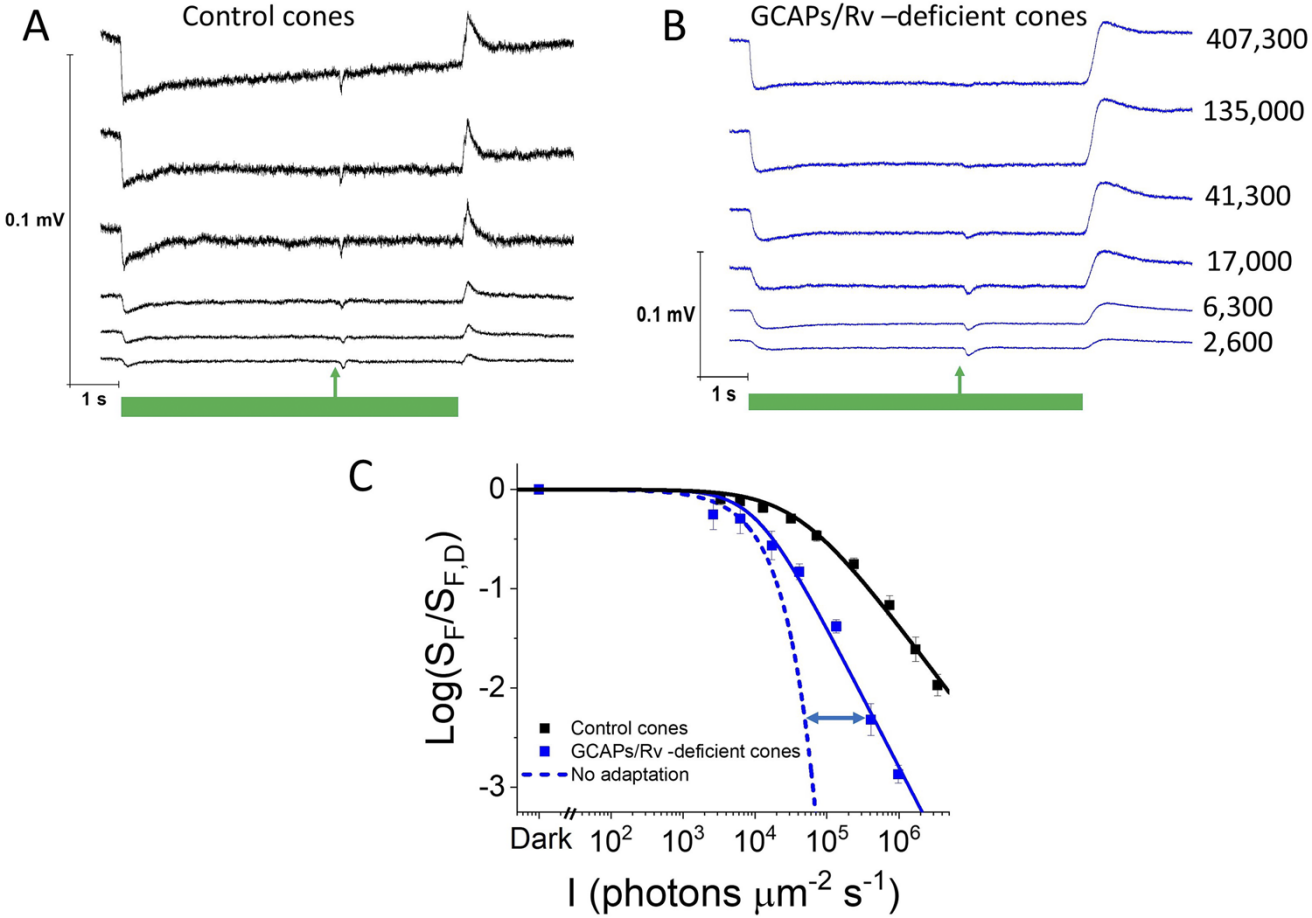
GCAPs/recoverin-deficient cones can adapt to background light. Responses of control (**A**) and GCAPs/recoverin-deficient (**B**) cones to steps of 505 nm light (indicated by green bars) from 2,620 up to 407,300 photons μm^−2^ s^−1^ (numbers on the right indicate the background light intensity, identical for A and B). A flash of light was delivered at 4.5 s after the step onset (arrow) to probe the sensitivity of cones (*S*_*F*_) during different backgrounds. (**C**) Normalized sensitivity (*S*_*F*_ /*S*_*F*,*D*_, where *S*_*F*,*D*_ is the sensitivity in darkness, (mean ± SEM) plotted as a function of background light intensity in photons μm^−2^ s^−1^ for control cones (black squares, n = 4 mice) and GCAPs/recoverin-deficient cones (blue squares, n = 3 mice). The smooth traces plot Eq. (2) with *I*_0_ = 39,600 μm^−2^ s^−1^ and *n* = 1.0 for control cones (black), and with *I*_0_ = 10,200 μm^−2^ s^−1^ and *n* = 1.4 for GCAPs/recoverin-deficient cones (blue). The blue dashed trace plots Eq. (3) calculated from the data measured from dark-adapted GCAPs/recoverin-deficient cones.

A recent study showed that GCAPs- and Rv-deficient mouse rods can still adapt to light^39^. However, the ability of cones lacking GCAPs and recoverin to modulate their phototransduction cascade has not been investigated. To address this question, we first recorded light responses of cones in isolated retinas to steps of light by using *ex vivo* ERGs. In control *Gnat1*^−/−^ cones, the initial hyperpolarization after the step onset was followed by partial recovery demonstrating the modulation of CNG channel current during steady background light (Fig. 2A). Exposure of cones lacking GCAPs and recoverin to identical steps of light produced larger responses as compared to those of control cones (Fig. 2A,B; note the different scale of the two y-axes). For example, the mean amplitude of step responses at steady state just before the test flash was flash was 2.6 ± 1 µV in control and 17 ± 1 µV at ~41,000 photons µm^−2^ s^−1^ background. This difference was highly significant (n = 3, p < 0.0005). Interestingly, despite lacking the two major components of their calcium feedback, *GCAPs*^−/−^ *Rv*^−/−^ *Gnat1*^−/−^ cones also showed a prominent recovery after the initial hyperpolarization following the onset of light step. Thus, even in the absence of both GCAPs and recoverin, mouse cones were able to regulate their CNG channel current and light-adapt during steady background light (Fig. 2B). To assess the light adaptation capacity of *GCAPs*^−/−^ *Rv*^−/−^ *Gnat1*^−/−^ cones, we probed their sensitivity in background lights of varying intensity by delivering a test flash 4.5 s after the onset of the background. As expected, the sensitivity of control *Gnat1*^−/−^ cones declined according to Weber-Fechner law (Fig. 2C, black, Table 1). In contrast, the sensitivity decline was steeper (i.e. n > 1 in Eq. 2, see Table 1) and appeared to be shifted to dimmer background lights in *GCAPs*^−/−^ *Rv*^−/−^ *Gnat1*^−/−^ cones (Fig. 2C, blue) than in controls (though, the change of I_0_ was not statistically significant, see Table 1). These results demonstrate that, as has been shown previously, GCAPs and recoverin are important for mouse cone light adaptation^32,46^. However, comparison of light adaptation in *GCAPs*^−/−^ *Rv*^−/−^ *Gnat1*^−/−^ cones to the expected decline of sensitivity in the absence of any adaptation mechanisms (Fig. 2C, blue dashed line) revealed that substantial light adaptation remains even in cones lacking both GCAPs and recoverin. This was confirmed by pair-wise comparison of the experimental data to the model data from six *GCAPs*^−/−^ *Rv*^−/−^ *Gnat1*^−/−^ mice at six different background light intensities ranging from 2,600 to 236,000 photons μm^−2^ s^−1^. This comparison demonstrated a significant difference between measured and calculated (Eq. 3) sensitivity above ~30,000 photons μm^−2^ s^−1^ (p < 0.01, paired Student t-test). The mechanisms for this GCAPs/Rv-independent light adaptation in mouse cones are currently not known. Here we investigated if the residual light adaptation capacity of GCAPs/Rv-deficient cones is mediated by Ca^2+^ -dependent and/or Ca^2+^-independent pathway(s).

### Modulation of CNG channel current and light response kinetics in cones by lowered Ca2+

The contribution of Ca^2+^-dependent mechanisms to the light adaptation of amphibian photoreceptors has been studied by several methods. Those methods include clamping Ca^2+^ concentration of a single photoreceptor to its dark-adapted or light-adapted level. This can be achieved for a brief time by removing Na^+^ and Ca^2+^ from the extracellular solution in darkness or during steady background light, or by manipulating Ca^2+^ levels in truncated photoreceptor outer segments that can be dialyzed with GTP or cGMP to study cGMP synthesis and hydrolysis, respectively^26,38,50–55^. These methods require rapid changes of extracellular environment of single cells or truncated outer segment compartments of photoreceptors, manipulations that are not well tolerated by fragile and small mouse rod or cone outer segments. Here, we adopted a different approach to force the cone phototransduction to its maximally light adapted state. We achieved that by lowering the extracellular Ca^2+^ concentration to drive intracellular Ca^2+^ level below that attained in bright light, thus fully engaging the Ca^2+^ feedback. To achieve that, we exposed isolated mouse retinas to low ~30 nM [Ca^2+^]_o_ during *ex vivo* ERG recordings to study how mouse cone physiology is affected under low Ca^2+^ environment. Assuming that the extrusion of Ca^2+^ via the cone Na^+^/Ca^2+^, K^+^ exchangers is linearly proportional to the intracellular Ca^2+^ concentration^56^, and that the conductance of Ca^2+^ is increased about 50% by low Ca^2+^ exposure in GCAPs^−/−^ Rv^−/−^ cones (see Fig. 4A below), it can be calculated that [Ca^2+^]_in_ under these conditions will drop from 250 nM in darkness in normal Ca^2+^^57^ to ~1 nM. Thus, at steady state in low Ca^2+^ exposure, the level of [Ca^2+^]_in_ in cones would be below its level even in very bright light, fully activating any Ca^2+^-mediated phototransduction feedbacks.

**Figure 3:**
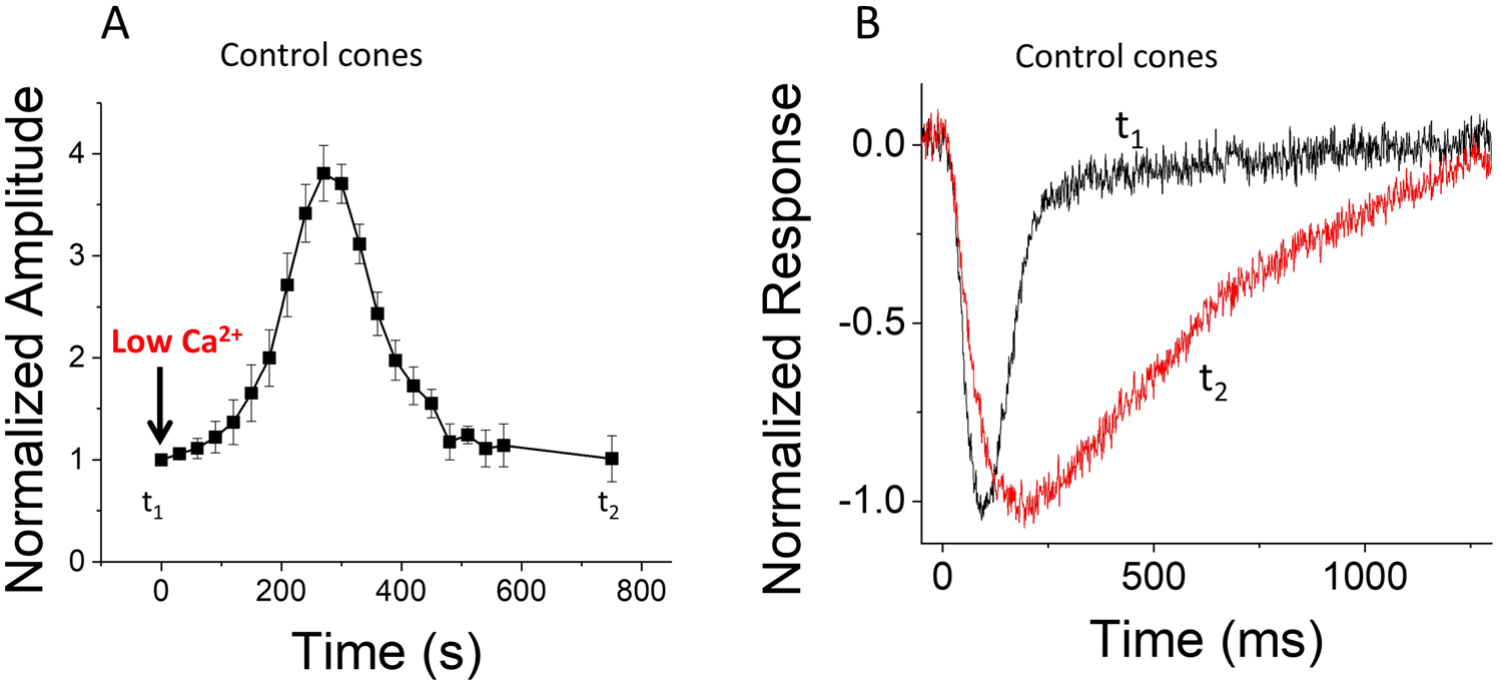
Low Ca^2+^ exposure causes large transient increase of *r*_*max*_ and deceleration of flash response kinetics in control mouse cones. (**A**) Normalized maximal cone response amplitudes (*r*_*max*_, mean ± SEM, n = 4 mice) to a saturating bright test flash plotted as a function of time after exposing the retina to low Ca^2+^ medium. Amplitudes have been normalized to *r*_*max*_ just before the low Ca^2+^ exposure. (**B**) Averaged normalized responses (mean, n = 4 mice) of control cones to a dim test flash producing a response with amplitude < 20% of *r*_*max*_ just before the low Ca^2+^ exposure (black) and about 10 min after the switch to low Ca^2+^ solution (red).

As has been shown previously for other photoreceptor types and/or species, the low Ca^2+^ exposure of retinas from control *Gnat1*^−/−^ mice led to a significant but only transient increase of the maximal light response amplitude (*r*_*max*_) in mouse cones (Fig. 3A; see also^10,39,58^). At steady state about 10 minutes after the switch to low Ca^2+^, the amplitude of *r*_*max*_ stabilized to approximately the same level as in normal Ca^2+^. However, comparison of dim flash responses in normal and low Ca^2+^ at steady state revealed a significant deceleration of response kinetics of cones caused by lowering Ca^2+^ (Fig. 3B), an effect similar to that observed previously in rod photoreceptors. Previous studies have suggested that the abnormally high cGMP in the photoreceptors exposed to low Ca^2+^ in darkness would not be well tolerated by the cells causing gradually declining response amplitudes^10,58^ and slowdown of their phototransduction^44,53,59^.

**Figure 4:**
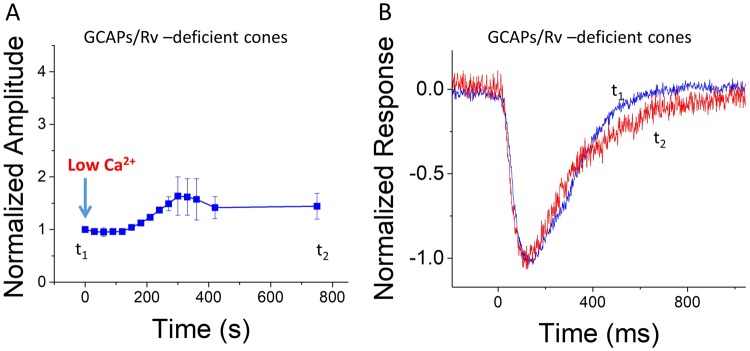
Low Ca^2+^ exposure causes moderate and stable increase of r_*max*_ in GCAPs/recoverin-deficient cones. (**A**) Normalized maximal cone response amplitudes (*r*_*max*_, mean ± SEM, n = 3 mice) to a saturating bright test flash plotted as a function of time after exposing the retina to low Ca^2+^ medium. Amplitudes have been normalized to *r*_*max*_ just before the low Ca^2+^ exposure. (**B**) Averaged normalized responses (mean, n = 3 mice) of GCAPs/recoverin-deficient cones to a dim test flash producing a response with amplitude < 20% of *r*_*max*_ just before the low Ca^2+^ exposure (black) and about 10 min after the switch to low Ca^2+^ solution (red).

In contrast to control cones, the cones of our *GCAPs*^−/−^ *Rv*^−/−^ *Gnat1*^−/−^ mice lack the GCAP-mediated acceleration of cGMP synthesis at low Ca^2+^. Therefore, low Ca^2+^ exposure of these cones would not be expected to produce increase in their cGMP concentration. Consistent with this hypothesis, the large transient increase of the maximal cone response amplitudes observed after the switch to low Ca^2+^ in cones from control *Gnat1*^−/−^ mice was absent in cones from *GCAPs*^−/−^ *Rv*^−/−^ *Gnat1*^−/−^ mice (Fig. 4A). However, we still observed about 50% increase of *r*_*max*_ by low Ca^2+^ exposure even in cones lacking GCAPs and recoverin (Fig. 4A), and these larger amplitudes remained stable for up to 30 minutes in most of the experiments. In striking contrast to the case in control cones, the kinetics of cone responses from *GCAPs*^−/−^ *Rv*^−/−^ *Gnat1*^−/−^ mice were not decelerated by the low Ca^2+^ exposure (Fig. 4B). The stability of response amplitudes and the lack of phototransduction deceleration indicate that in the absence of GCAPs-mediated acceleration of cGMP synthesis low Ca^2+^ exposure does not induce any toxic effects. This allowed us to use this low Ca^2+^ method in *GCAPs*^−/−^ *Rv*^−/−^ *Gnat1*^−/−^ mice to study the contribution of Ca^2+^-dependent and any possible Ca^2+^-independent mechanisms to the light adaptation of mouse cones in the absence of GCAPs- and recoverin-mediated regulation.

### Cones lacking both GCAPs and recoverin can light adapt via both Ca^2+^-dependent and Ca^2+^-independent mechanisms

**Figure 5:**
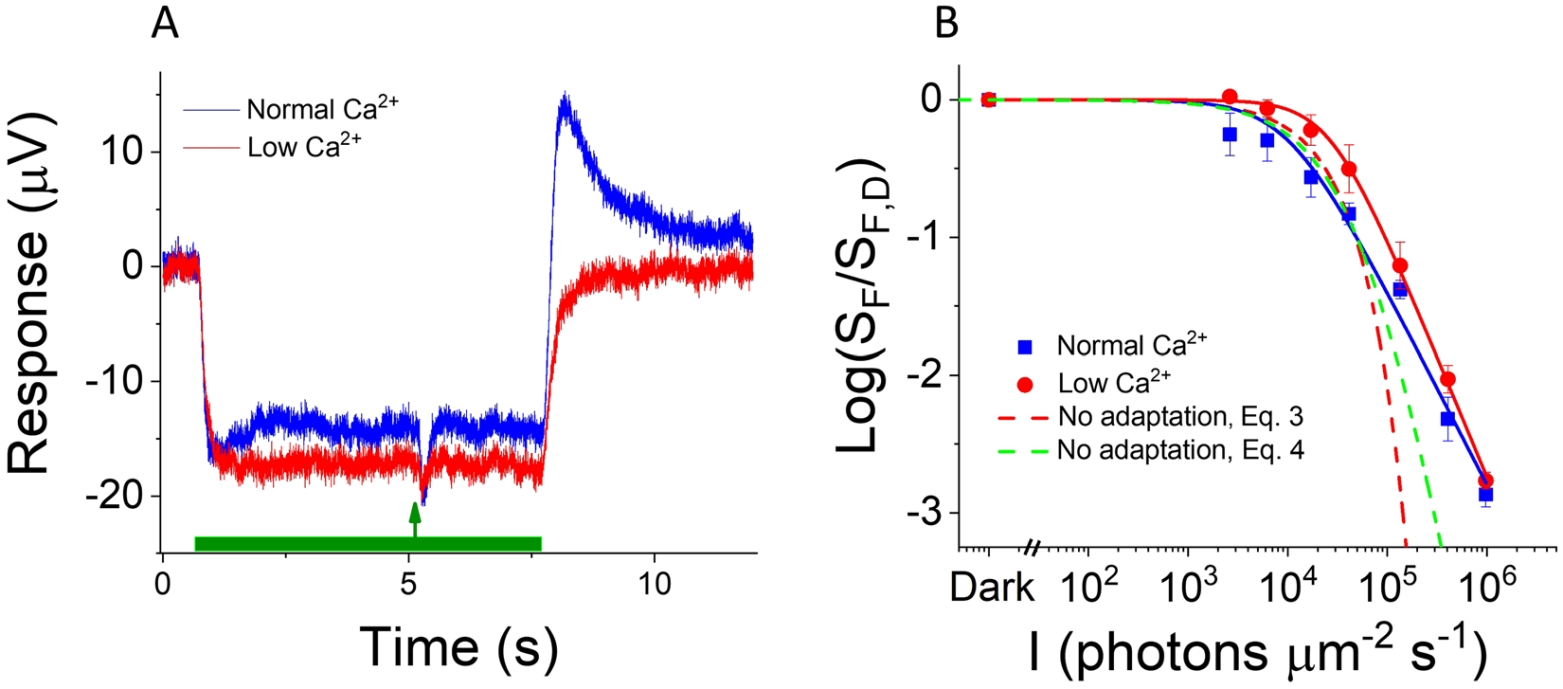
Ca^2+^-dependent and Ca^2+^-independent light adaptation mechanisms contribute to the light adaptation capacity of cones lacking both GCAPs and recoverin. (**A**) A response to a step of light (I = 17,100 photons μm^−2^ s^−1^) superimposed with a test flash (arrow) recorded from GCAPs/recoverin-deficient cones in normal (blue) and low (red) Ca^2+^ (same retina). The test flash strength was 1,600 and 570 photons μm^−2^ in normal and low Ca^2+^, respectively. (**B**) Sensitivity of cones (S_F_) normalized to the sensitivity in darkness (S_F,D_) plotted as function of background light intensity (I) for GCAPs/recoverin-deficient cones in normal (blue squares) and low (red squares) Ca^2+^. Smooth lines plot Eq. (2) with I_0_ = 10,200 photons μm^−2^ s^−1^ and n = 1.4 in normal Ca^2+^ (blue) and with I_0_ = 27,000 photons μm^−2^ s^−1^ and n = 1.7 in low Ca^2+^ (red). The dashed red and green traces plot Eqs (3) and (4), respectively, with parameter values calculated from dark-adapted responses of these cones in low Ca^2+^. Sensitivity data (mean ± SEM) in (**B**) is from 3 GCAPs^−/−^ Rv^−/−^ Gnat1^−/−^ mice for which we used identical background light intensities. The theoretical traces plot the mean values for the same 3 GCAPs^−/−^ Rv^−/−^ Gnat1^−/−^ mice. For comprehensive statistical analysis, see Table 1 and text.

Finally, we evaluated the contribution of Ca^2+^-dependent and Ca^2+^-independent mechanisms of light adaptation in cones already devoid of their two known feedback mechanisms, *via* GCAPs and recoverin. To do that, we compared the light adaptation of *GCAPs*^−/−^ *Rv*^−/−^ *Gnat1*^−/−^ mouse cones in normal Ca^2+^, where a potential dynamic light-induced Ca^2+^-dependent mechanism would be functional, and in low Ca^2+^, where any residual Ca^2+^ feedback would be driven to a fully light-adapted state. Exposure of cones lacking GCAPs and recoverin to a step of background light in normal Ca^2+^ produced an initial hyperpolarization, followed by partial recovery indicative of adaptation of the cone phototransduction cascade (Fig. 5A, blue). As shown in Fig. 2C, the decline in their sensitivity in backgrounds of increasing intensity was steeper than in control cones (i.e. n increased upon deletion of GCAPs and Rv, Table 1), but still well above what would be expected in the lack of any adaptation (Fig. 5B blue). These results suggest the existence of a feedback mechanism contributing to mouse cone light adaptation even in the absence of the known GCAPs- and recoverin-mediated pathways.

When GCAPs/recoverin-deficient cones were exposed to a step of light in low Ca^2+^, the rapid partial recovery in their response was no longer observable (Fig. 5A, red). In addition, the sensitivity of these cones in low Ca^2+^ started to decline at somewhat brighter background light intensities (*I*) than in normal Ca^2+^ (i.e. I_0_ increased in low Ca^2+^, see Table 1). However, the subsequent decline was steeper (i.e. the steepness parameter n was larger in low Ca^2+^, see Table 1) so that the sensitivities under normal and low Ca^2+^ appeared to converge at brighter background light intensities (Fig. 5B, blue and red squares and solid lines). To quantitatively confirm convergence of the sensitivity in normal and low Ca^2+^, we performed statistical comparison of the normalized sensitivity data at five different background light intensities ranging from ~6,000 to 400,000 photons μm^−2^ s^−1^ using paired Student t-test. The analysis from six *GCAPs*^−/−^ *Rv*^−/−^ *Gnat1*^−/−^ mice demonstrated that the p-value, indicating significant difference, gradually increased from 0.01 to 0.01, 0.03, and 0.04 for the four dimmest backgrounds, and finally reached 0.13, indicating no significant difference, for the brightest background. To evaluate whether GCAPs/recoverin-deficient cones in low Ca^2+^ undergo any light adaptation, we calculated the predicted drop of sensitivity as a function of *I* under low Ca^2+^ (Fig. 5B, dashed red and green lines). Although partially suppressed by the pharmacological reduction in Ca^2+^, these cones appeared to have a wider dynamic range and performed better than expected by the theoretical “no adaptation” model. To confirm the presence of light adaptation under low Ca^2+^, we compared measured sensitivities and those calculated by Eq. 4 from six *GCAPs*^−/−^ *Rv*^−/−^ *Gnat1*^−/−^ mice at five different background light intensities ranging from ~6,000 to 400,000 photons μm^−2^ s^−1^. The sensitivity calculated by the model was lower at each of the five backgrounds (paired Student t-test, p < 0.05). We also analyzed if background light shortened the time-to-peak of a dim flash response of a dark adapted DKO cone under low Ca^2+^ perfusion. The time-to-peak shortened 38 ± 6% (mean ± SEM) under background of ~150,000 photons μm^−2^ s^−1^ as compared to that in darkness, a difference found to be highly significant (n = 6, p < 0.005, paired Student t-test). Further increase of background light intensity did not cause a further acceleration of response kinetics. Although voltage-gated channels and capacitive currents can also shape the ERG signals^60–62^, the robust Weber adaptation observed in our experiments indicates that these findings are not affected by voltage-dependent or capacitive currents. We conclude that, in striking contrast to mouse rods, mammalian cones appear to have a unique mechanism for Ca^2+^-independent light adaptation.

## Discussion

Light-induced decrease in the photoreceptor outer segment Ca^2+^ concentration is a signal that mediates several feedback mechanisms via Ca^2+^ sensor proteins GCAPs^28,29,46^, recoverin^32,34,40^ and calmodulin/CNG modulin^35,37^ to modulate cGMP synthesis, active visual pigment lifetime, and CNG channels conductance, respectively. Many studies have indicated that Ca^2+^ is both necessary and sufficient for the light adaptation of amphibian photoreceptors, i.e. without Ca^2+^ feedbacks the responses of dark-adapted photoreceptors to single photons would be summed linearly leading to a rapid exponential saturation of these photoreceptors at very dim background light levels^26,27,54^. Some studies have suggested that Ca^2+^-independent adaptation mechanism(s) could also contribute to the light adaptation of amphibian rods^63–66^. However, Nikonov *et al*.^63^ have argued that in salamander rods the acceleration of response shut-off caused by background light is due to a trivial Ca^2+^-independent acceleration of cGMP turnover following activation of PDE by light and does not require existence of any specific feedback pathway. Furthermore, even if this increase in the steady state cGMP hydrolysis rate is taken into account, a significant amount of light adaptation persists even in mouse rods lacking GCAPs- and recoverin-mediated feedbacks^39^. In amphibians, the contribution of Ca^2+^-dependent mechanisms to the light adaptation capacity of photoreceptors has been studied mainly by manipulating the ionic environment around photoreceptor outer segments to prevent the light-induced change in their Ca^2+^ concentration (“Ca^2+^ clamp” method^26,27,38,52,54^). On the other hand, the role of different molecular mechanisms to the light adaptation of mouse, Xenopus and zebrafish photoreceptors has been studied by combining genetic and physiology approaches^2,67,68^. However, mouse photoreceptors are small and fragile making it very challenging to perform physiological recordings from their rods or cones in isolation. Thus, Ca^2+^ clamp experiments have not been performed successfully from mouse rod or cone photoreceptors.

As an alternative approach, light-induced decrease in Ca^2+^ concentration could be mimicked by reducing the extracellular Ca^2+^ that would drive the intracellular Ca^2+^ concentration to a lower level. However, in contrast to the light-induced acceleration of flash response kinetics, an exposure of photoreceptors to a very low Ca^2+^ in darkness causes a significant deceleration of flash response kinetics^44,53,54,59^. In addition, after the initial large increase, the CNG channel current gradually decreases following exposure to low Ca^2+^^10,39^. A light-induced drop in Ca^2+^ also causes acceleration of cGMP hydrolysis so that cGMP concentration or CNG channel current do not exceed the values observed in dark-adapted photoreceptors. In contrast, exposure to low Ca^2+^ in darkness is expected to increase cGMP concentration due to the GCAP-mediated feedback on guanylyl cyclase activity. The hypothesis that high cGMP is causing the anomalous effects of low Ca^2+^ exposure in mouse rods (directly or indirectly) was tested recently by exposing rods lacking the GCAP-mediated feedback on cyclase to a very low (about 30 nM) [Ca^2+^]^39^. In striking contrast to wild type rods, the flash responses of *GCAPs*^−/−^ rods were accelerated by low Ca^2+^ exposure. Thus, low Ca^2+^ exposure in the absence of GCAPs-mediated increase of cGMP concentration in rods is a viable approach for dissecting the contribution of Ca^2+^-dependent and Ca^2+^-independent mechanisms of phototransduction and light adaptation. Here, we applied this approach to study light adaptation in mouse cones that lack Ca^2+^ feedbacks mediated by GCAPs and recoverin by comparing the light adaptation of *GCAP*^−/−^ *Rv*^−/−^ *Gnat1*^−/−^ mouse cones between normal (1.2 mM) and low (~30 nM) extracellular [Ca^2+^]. As in wild type rods, cones expressing GCAPs also showed a significant but only transient increase of their *r*_*max*_ after exposure to low Ca^2+^ (Fig. 3A). Furthermore, similarly to mouse rods, the kinetics of their flash responses slowed down (Fig. 3B). However, the value of *r*_*max*_ of GCAPs/recoverin-deficient cones increased only moderately and remained relatively stable during low Ca^2+^ exposure (Fig. 4A). Importantly, the kinetics of flash responses also did not slow down by low Ca^2+^ exposure in these cones (Fig. 4B).

The Ca^2+^ concentration in the outer segments of photoreceptors is maintained at about 10,000-fold lower level than outside the cells by active extrusion of Ca^2+^ via Na^+^/Ca^2+^, K^+^ exchangers (NCKX,^25,69–71^). Thus, it is expected that during our low Ca^2+^ treatment, outer segment Ca^2+^ would be below the ~20 nM attained in bright light^57^ and well below the operating range of the potential Ca^2+^ feedback mechanisms. For example, the affinity of Ca^2+^ to mouse GCAP1 is ~130 nM and to GCAP2 ~50 nM^72^. Consequently, at ~10 nM almost all of the GCAPs would be in a Ca^2+^-free form and any further reduction caused by light would not have any modulatory effect on light adaptation. Indeed, the converging sensitivity of cones between normal and low Ca^2+^ when background light intensity increases suggests that during our low Ca^2+^ exposure, the cones are at physiological fully light-adapted state with regards to their Ca^2+^ feedback mechanisms (Fig. 5B).

Cones are much less sensitive to light and can operate at significantly brighter ambient illumination levels as compared to rods. A recent review summarized nicely what is known about differences of the phototransduction proteins and their expression levels between rod and cone photoreceptors, and how much these differences could contribute to the sensitivity difference of mouse rods and cones^1^. Studies comparing fish and avian rods and cones have demonstrated several differences in the activity and expression levels of rod- and cone-specific phototransduction proteins that could potentially explain the physiological differences between rods and cones (for review, see^73^). Thus, the cone-specific kinase GRK7 (though not expressed in mouse photoreceptors) is more active and highly expressed as compared to rod GRK1^13^. The active form of chicken cone visual pigment, Meta II, decays 50 times faster than rhodopsin^15^. Cone pigments are also less stable^22,74^ and noisier than rod pigment^75,76^. In addition, the enzyme RGS9 known to accelerate inactivation of active transducing-phosphodiesterase complex, the cGMP-synthesizing enzyme guanylyl cyclase, as well as arrestins are expressed at higher levels in carp cones than in rods^14,16,17^. Quantification of expression levels of phototransduction proteins in mouse cones is challenging due to their very small percentage in the mouse retina. Many studies have tried to assess the different properties of cone vs. rod isoforms of visual pigments, G proteins and PDE6 by expressing the cone isoforms in the rod photoreceptors^21,77–81^. These studies suggest that differences in the activity of rod and cone phototransduction enzymes may contribute to the difference in the sensitivity of mouse rods and cones but the molecular origin of the sensitivity difference between mammalian rods and cones remains poorly understood^1^. It is known that the amphibian outer segment Ca^2+^ concentration changes more rapidly and over wider range in cones than in rods^82,83^. This could translate into more efficient regulation of cone phototransduction sensitivity by background light via Ca^2+^ feedback mechanisms. However, the main Ca^2+^ feedback, mediated by GCAPs, contributes similarly to the regulation of sensitivity and light adaptation in mouse rods and cones^46^. Although recoverin appears to contribute slightly more to the regulation of cone than rod phototransduction, its role in the mouse phototransduction and light adaptation is rather small both in rods and cones and certainly cannot explain the large differences between these cells^32,40^. A recent study demonstrated that rods lacking both GCAPs and recoverin still can light adapt via some other Ca^2+^-dependent mechanism^39^. Here, we show the cones of these mice also have a Ca^2+^-dependent light adaptation component. However, overall this residual Ca^2+^ feedback in GCAPs/recoverin-deficient mouse photoreceptors is rather small and its magnitude is not larger in cones than in rods (Fig. 5B and^39^). On the other hand, our results reveal a substantial Ca^2+^-independent light adaptation component in mouse cones that is not present in rods. This adaptation is apparent even when the upregulation of cGMP flux by the background light is taken into consideration (Fig. 5B). Thus, surprisingly it appears that the difference between the adaptation capacities of mammalian rod and cone photoreceptors may not be explained by Ca^2+^-dependent mechanism. Instead, our findings suggest that future efforts in understanding the functional differences between rods and cones should be focused on identifying Ca^2+^-independent molecular mechanism(s).

Our results demonstrated a robust Ca^2+^-dependent light adaptation mechanism even in the absence of GCAPs and recoverin in mouse cones (Fig. 5B). We observed about 50% increase of *r*_*max*_ by low Ca^2+^ exposure in *GCAPs*^−/−^ *Rv*^−/−^ *Gnat1*^−/−^ cones, indicating that some Ca^2+^-dependent mechanism can potentiate the CNG channel current in these cones (Fig. 4A). On the other hand, lowering Ca^2+^ did not affect much the response kinetics (Fig. 4B). A very small effect of low Ca^2+^ exposure to response kinetics in *GCAPs*^−/−^ *Rv*^−/−^ *Gnat1*^−/−^ cones (and rods) suggest that the Ca^2+^ feedback is not modulating cGMP hydrolysis or synthesis rate (Fig. 4B). Another potential mechanism for the observed Ca^2+^ feedback is the modulation of CNG channels. This modulation may be via a recently discovered CNG modulin or its mammalian homolog Eml1^35,36^. Future studies combining genetics and cone physiology will be able to test these hypotheses.

Our results clearly demonstrate the robust function of a Ca^2+^-independent mechanism for light adaptation in mouse cones (Fig. 5B) that is not present in rods^39^. One trivial explanation for this residual light adaptation in cones is a simple light-induced acceleration of cGMP hydrolysis. The reduction in free cGMP will result in easier and faster subsequent change of the fractional cGMP concentration even without upregulating cGMP synthesis^63^. However, using a phototransduction model with all active feedbacks removed to estimate the drop of sensitivity^45^ reveals that a significant amount of light adaptation persists in GCAPs/recoverin-deficient cones even in low Ca^2+^ (Fig. 5B, dashed green trace). Comparing the flash responses in darkness and under various backgrounds in their cones under low Ca^2+^ exposure shows that background light can progressively accelerate light response termination (see Results). Thus, it is possible that the novel Ca^2+^-independent mechanism identified here accelerates cGMP synthesis rate. In principle, this could be related to a recently suggested bicarbonate-dependent modulation of guanylyl cyclase although it is not clear whether bicarbonate concentration can be modulated in background light-dependent manner^84^. Another possible mechanism for the Ca^2+^-independent adaptation could be a recently discovered pathway acting via IGF-1 and all-*trans*-retinol to regulate the rod CNG channels^66,85^. This mechanism causes potentiation (increase of light sensitivity) of rod response amplitudes in background light. Whether it can contribute to the light adaptation in mouse cones and explain the Ca^2+^-independent light adaptation observed here will be an interesting subject for future studies.

## Data Availability

The datasets generated and analysed during the current study are available from the corresponding author on reasonable request.
